# Gallbladder cancer mimicking perihilar cholangiocarcinoma—considerable rate of postoperative reclassification with implications for prognosis

**DOI:** 10.1186/s12957-023-03171-x

**Published:** 2023-09-11

**Authors:** Lynn E. Nooijen, Maria Gustafsson-Liljefors, Joris I. Erdmann, Melroy A. D’Souza, Stefan Gilg, Christina Villard, Hannes Jansson

**Affiliations:** 1 Department of Surgery, Cancer Center Amsterdam, Amsterdam University Medical Center, Amsterdam, the Netherlands; 2 Division of Surgery and Oncology, Department of Clinical Science, Intervention and Technology, Karolinska Institutet, Karolinska University Hospital, Stockholm, Sweden; 3 Gastroenterology and Rheumatology Unit, Department of Medicine Huddinge, Karolinska Institutet, Karolinska University Hospital, Stockholm, Sweden; 4 Division of Transplantation Surgery, Department of Clinical Science, Intervention and Technology, Karolinska Institutet, Karolinska University Hospital, Stockholm, Sweden

**Keywords:** Perihilar cholangiocarcinoma, Preoperative diagnosis, Postoperative prognosis, Biliary tract cancer, Gallbladder cancer

## Abstract

**Background:**

For some patients undergoing resection under the suspicion of a perihilar cholangiocarcinoma (pCCA), postoperative diagnosis may differ from the preoperative diagnosis. While a postoperative finding of benign bile duct stricture is known to affect 3–15% of patients, less has been described about the consequences of finding other biliary tract cancers postoperatively. This study compared pre- and postoperative diagnoses, risk characteristics, and outcomes after surgery for suspected pCCA.

**Methods:**

Retrospective single-center study, Karolinska University Hospital, Stockholm, Sweden (January 2009–May 2017). The primary postoperative outcome was overall survival. Secondary outcomes were disease-free survival and postoperative complications. Survival analysis was performed by the Kaplan–Meier method.

**Results:**

Seventy-one patients underwent resection for suspected pCCA. pCCA was confirmed in 48 patients (68%). Ten patients had benign lesions (14%), 2 (3%) were diagnosed with other types of cholangiocarcinoma (CCA, distal *n* = 1, intrahepatic *n* = 1), while 11 (15%) were diagnosed with gallbladder cancer (GBC). GBC patients were older than patients with pCCA (median age 71 versus 58 years, *p* = 0.015), with a large proportion of patients with a high tumor extension stage (≥ T3, 91%).

Median overall survival was 20 months (95% CI 15–25 months) for patients with pCCA and 17 months (95% CI 11–23 months) for patients with GBC (*p* = 0.135). Patients with GBC had significantly shorter median disease-free survival (DFS), 10 months (95% CI 3–17 months) compared 17 months (95% CI 15–19 months) for patients with pCCA (*p* = 0.010).

**Conclusions:**

At a large tertiary referral center, 15% of patients resected for suspected pCCA were postoperatively diagnosed with GBC. Compared to patients with pCCA, GBC patients were older, with advanced tumors and shorter DFS. The considerable rate of re-classification stresses the need for improved preoperative staging, as these prognostic differences could have implications for treatment strategies.

## Background

A reliable pre-operative diagnosis for patients presenting with a resectable obstructing hilar mass remains a clinical challenge. Most patients will turn out to have a perihilar cholangiocarcinoma (pCCA). However, a postoperative diagnosis of benign bile duct stricture is known to affect approximately 3–15% of patients resected on suspicion of perihilar cholangiocarcinoma in current series [1, 2]. Less has been described about the change of diagnosis to other subtypes of biliary tract cancers (BTC) on postoperative histopathology. This may be especially relevant as each subtype of BTC has a different prognosis and response to oncological therapy. The aim of this study was therefore to compare pre- and postoperative diagnoses and outcomes for patients undergoing hepatobiliary resection for suspected pCCA.

## Methods

Patients undergoing primary resection for suspected pCCA at Karolinska University Hospital (Stockholm, Sweden), a tertiary referral center, in the period January 2009 to May 2017 were included in this study. Data were retrospectively collected from local quality registries and the electronic health records. The study was approved by the Regional Ethical Review Board and Swedish Ethical Review Authority.

The primary postoperative outcome was overall survival, calculated from the date of surgery. Secondary outcomes were disease-free survival calculated from the date of surgery, postoperative complications according to the Clavien-Dindo classification [3], postoperative liver failure according to the applied Balzan 50:50 criteria [4, 5] (bilirubin > 50 micromol/L and prothrombin-international normalized ratio > 1.5 on postoperative day 5) and mortality (within 90 days or in-hospital if length of stay exceeding 90 days). Follow-up time was calculated according to the reverse Kaplan–Meier method. Clinicopathological data collected included age, gender, body mass index (BMI), American Society of Anesthesiologists physical status classification (ASA), diagnosis of primary sclerosing cholangitis, Bismuth-Corlette classification [6], the type and extent of resection (major resection defined as including three or more hepatic segments), preoperative portal vein embolization, preoperative biliary drainage, preoperative cholangitis requiring additional invasive intervention, preoperative Glasgow Prognostic Score (GPS, C-reactive protein increase, and/or hypoalbuminemia) [7], preoperative plasma bilirubin concentration, postoperative pathological tumor extension (T), lymph node metastasis (N1), lymphovascular invasion (LV1), perineural (Pn1) invasion, and microscopically tumor positive resection margin defined as invasive cancer within 1 mm of the margin [8] (R1). The preoperative diagnosis of pCCA was based on clinical aspects, cross-sectional radiological imaging, and cholangiography findings with consensus made during the multidisciplinary tumor board. Cholangioscopy or intraductal ultrasound was not routinely performed as part of the preoperative diagnostic work-up. Portal vein embolization was performed if the future liver remnant volume was below 30%, preceded by biliary decompression in jaundiced patients. Postoperative diagnosis was registered as determined by routine histopathological evaluation of the resected specimen in accordance with AJCC/TNM 7th edition guidelines and the College of American Pathologists, with gallbladder cancer (GBC) diagnosed as tumors extending from the gallbladder or cystic duct and pCCA diagnosed in tumors arising in the right-left or common hepatic ducts [9].

## Results

**Fig. 1:**
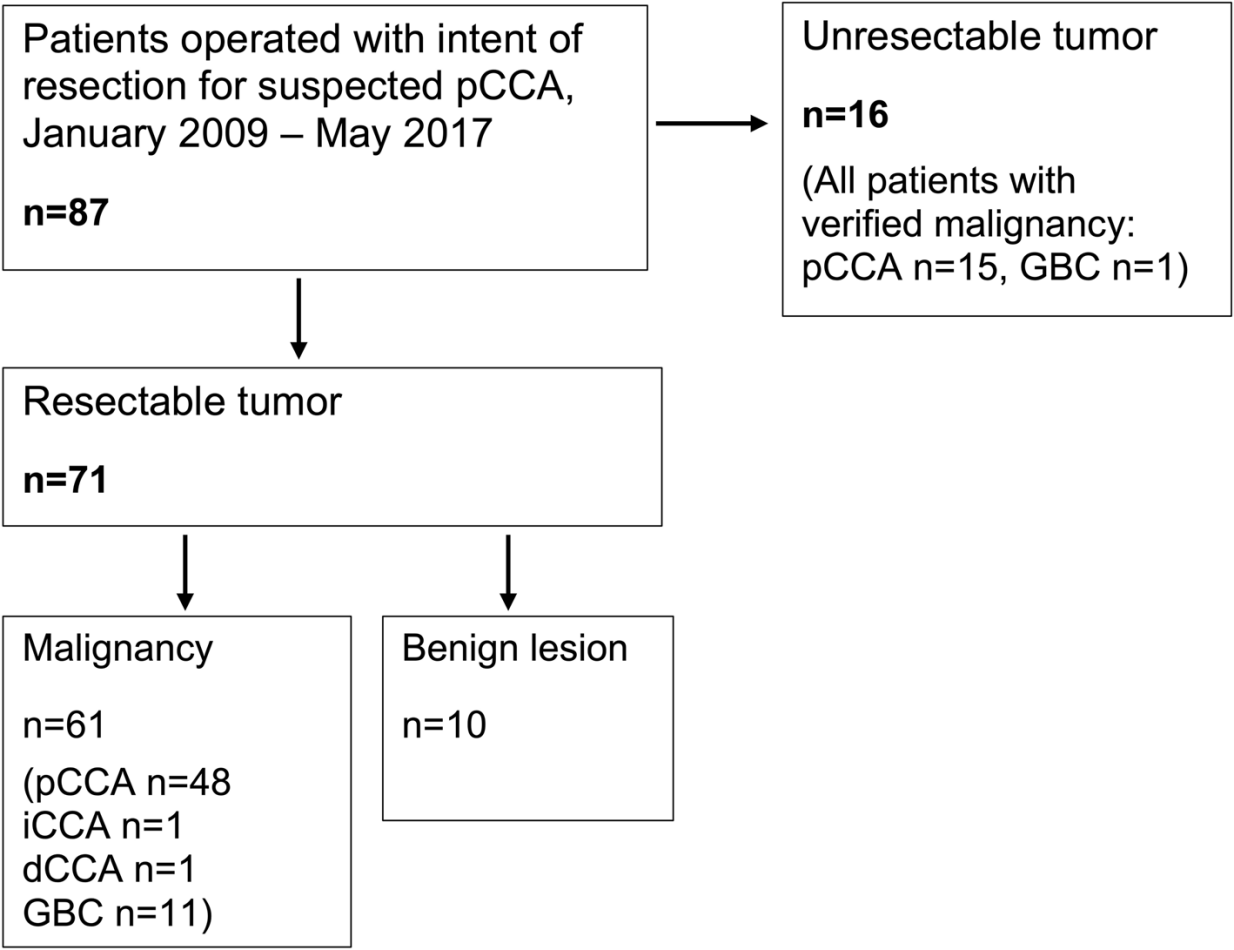
Study inclusion flow chart. dCCA: distal cholangiocarcinoma; GBC: gallbladder cancer; iCCA: intrahepatic cholangiocarcinoma; pCCA: perihilar cholangiocarcinoma

Seventy-one patients underwent resection with a preoperative clinical suspicion of pCCA and were included, while during the study period 16 patients underwent surgical exploration and were diagnosed with an unresectable tumor (Fig. 1). The median age was 62 years (IQR 51–71 years). Median follow-up was 71 months (IQR 58–83 months).

The pre-operative suspicion of pCCA was postoperatively confirmed in 48 out of the 71 patients (68%). Ten patients were found to have benign lesions (14%). Two patients (3%) were diagnosed with other subtypes of CCA showing perihilar engagement (distal CCA in one patient, intrahepatic CCA in one patient). Surprisingly, 11 patients (15%) were diagnosed with GBC.

Demographic and clinicopathological characteristics for patients with pCCA and GBC are presented in Table 1. Patients with a postoperative diagnosis of GBC were older, with a high proportion of tumors with extension T3 or above and less frequently underwent major resection compared to patients with pCCA. All GBC patients had a preoperative Bismuth-Corlette classification of I, II, or IIIa, i.e., common hepatic duct lesions or lesions extending into the right hepatic sectional duct confluence. All patients with a preoperative Bismuth-Corlette classification of IIIb or IV, i.e., lesions involving the left hepatic sectional duct confluence or the bilateral sectional duct confluences, were postoperatively diagnosed with pCCA. All patients with a Bismuth-Corlette type IV lesion were operated with an extended right hemihepatectomy. Portal vein resections were performed in 23% of pCCA patients and 18% of GBC patients (*P* = 1.000). No patient underwent an arterial resection. Postoperative short-term outcomes are presented in Table 2. For patients with benign lesions, median age was 60 years (IQR 43–73 years), median overall survival was not reached and 5-year overall survival was 70%. For patients with confirmed biliary tract cancer, median overall survival was 19 months (95% CI 14–24 months) and 5-year overall survival was 21%.

**Table 1:** Clinicopathological characteristics

Variable	All (pCCA and GBC)	pCCA confirmed (*n* = 48)	GBC (*n* = 11)	*P* value
Age, md (IQR)	62 (53–69)	58 (50–68)	71 (61–75)	0.015^b^
Gender				0.176^c^
Women, *n* (%)	25 (42)	18 (38)	7 (64)	
Men, *n* (%)	34 (58)	30 (63)	4 (36)	
BMI, md (IQR)	24 (22–27)	24 (22–27)	25 (24–28)	0.330^b^
PSC (yes), *n* (%)	7 (12)	7 (15)	0 (0)	0.328^c^
ASA ≥ 3 (missing data *n* = 1), *n* (%)	18 (31)	14 (30)	4 (36)	0.724^c^
Bilirubin, micromol/L (missing data *n* = 3), md (IQR)	12 (8–20)	12 (8–20)	16 (10–21)	0.410^b^
GPS ≥ 1 (missing data *n* = 11), *n* (%)	39 (81)	34 (83)	5 (71)	0.601^c^
Radiology				0.297^c^
CT only, *n* (%)	38 (64)	29 (60)	9 (82)	
CT + MRI, *n* (%)	21 (36)	19 (40)	2 (18)	
Preoperative biliary drainage (ERC/PTC), *n* (%)	56 (95)	46 (96)	10 (91)	0.260^c^
Preoperative cholangitis, *n* (%)	10 (17)	9 (19)	1 (9)	0.669^c^
PVE, *n* (%)	18 (31)	16 (33)	2 (18)	0.476^c^
Bismuth-Corlette class, *n* (%)				0.004^c^
I	6 (10)	3 (6)	3 (27)	
II	13 (22)	7 (15)	6 (55)	
IIIa	27 (46)	25 (52)	2 (18)	
IIIb	7 (12)	7 (15)	0	
IV	6 (10)	6 (13)	0	
Type of resection				0.028^c^
Right hemihepatectomy	4 (7)	4 (8)	0	
Extended right hemihepatectomy	38 (64)	31 (65)	7 (64)	
Left hemihepatectomy	3 (5)	3 (6)	0	
Extended left hemihepatectomy	6 (10)	6 (13)	0	
Bisegmentectomy 4b-5	3 (5)	0	3 (27)	
Isolated extrahepatic bile duct resection	5 (8)	4 (8)	1 (9)	
Portal vein resection, *n* (%)	13 (22)	11 (23)	2 (18)	1.000^c^
T ≥ 3, *n* (%) ^a^	26 (45)	16 (33)	10 (91)	
T4, *n* (%) ^a^	5 (8)	3 (6)	2 (18)	
N1 (missing data *n* = 3), *n* (%)	34 (61)	25 (56)	9 (82)	0.171^c^
Pn1 (missing data *n* = 2), *n* (%)	55 (96)	44 (96)	11 (100)	1.000^c^
LV1 (missing data *n* = 2), *n* (%)	49 (86)	38 (83)	11 (100)	0.332^c^
R1,* n* (%)	50 (85)	42 (88)	8 (73)	0.347^c^
Adjuvant therapy (missing data *n* = 11),* n* (%)	4 (8)	4 (10)	0 (0)	1.000^c^

*ASA* American Society of Anesthesiologists physical status classification, *BMI* Body mass index, *CT* Computerized tomography, *ERC* Endoscopic retrograde cholangiography, *GBC* Gallbladder cancer, *GPS* Glasgow Prognostic Score, *IQR* Interquartile range, *LV1* Lymphovascular invasion, *MRI* Magnetic resonance imaging, *N1* Lymph node metastasis; *pCCA* perihilar cholangiocarcinoma, *PSC* Primary sclerosing cholangitis, *PTC* Percutaneous transhepatic cholangiography, *PVE* Portal vein embolization, *R1* Microscopically positive resection margin, *T* ≥ *3* tumor extension 3–4. *T4* tumor extension 4

^a^According to the separate AJCC/TNM criteria for perihilar cholangiocarcinoma and gallbladder cancer respectively

^b^Mann-Whitney *U* test

^c^Fisher’s exact test

**Table 2:** Postoperative short-term outcomes

Variable	pCCA confirmed (*n* = 48)	GBC (*n* = 11)	*P* value
Major postoperative complications (Clavien-Dindo ≥ 3a), *n* (%)	28 (58)	7 (64)	1.000^a^
Postoperative liver failure, *n* (%)	8 (17)	3 (27)	0.413^a^
Mortality (within 90 days or in hospital), *n* (%)	6 (13)	2 (18)	0.635^a^

*GBC* Gallbladder cancer, *pCCA* perihilar cholangiocarcinoma

^a^Fisher’s exact test

**Fig. 2:**
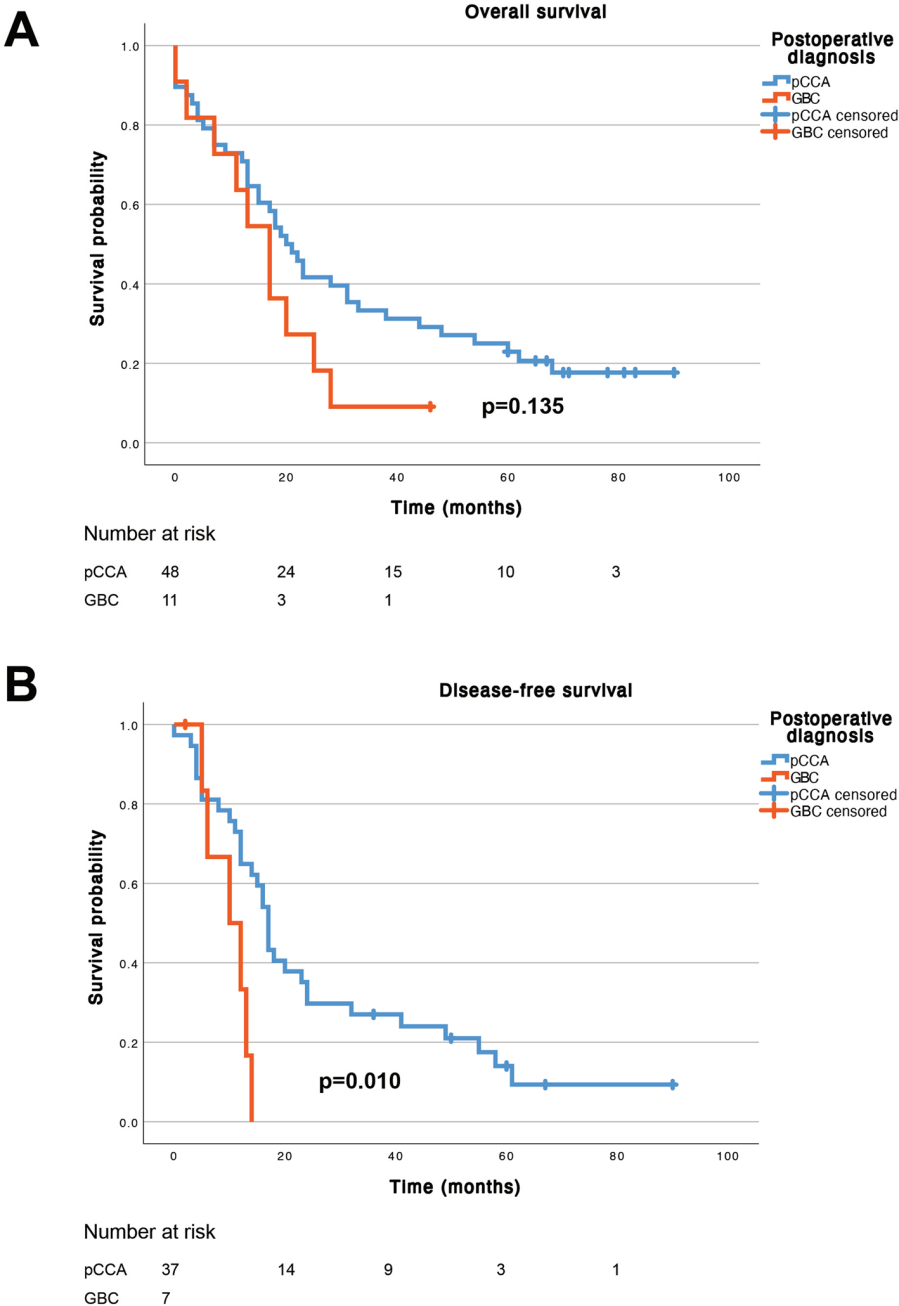
Postoperative overall survival (**A**) and disease-free survival (**B**). Patients with confirmed pCCA blue lines, patients with postoperative diagnosis of GBC red lines. pCCA: perihilar cholangiocarcinoma; GBC: gallbladder cancer. *P* values by log-rank test

In survival analysis, comparing patients with confirmed pCCA and patients with a postoperative finding of GBC, no significant difference was seen in overall survival (*p* = 0.135, Fig. 2A). Median overall survival was 20 months (95% CI 15–25 months) for patients with pCCA, and 17 months (95% CI 11–23 months) for patients with a postoperative diagnosis of GBC. Three-year overall survival was 33% for patients with pCCA and 9% for patients with GBC. Recurrence status was available for 46 out of 61 patients with a malignancy (75%). Comparing disease-free survival, patients with confirmed pCCA had a median disease-free survival of 17 months (95% CI 15–19 months), while patients with a postoperative diagnosis of GBC had a median disease-free survival of 10 months (95% CI 3–17 months) (*p* = 0.010, Fig. 2B).

The frequency of chemotherapeutic treatment after recurrence was similar in patients with pCCA and GBC (58% and 50% respectively, *p* = 1.000, missing data for recurrence treatment *n* = 12). While not a statistically significant difference, two patients with pCCA underwent re-resection for isolated local recurrence, compared to no patient with GBC (*p* = 1.000). Isolated local recurrence occurred in 12 patients (43%) with pCCA and two patients (33%) with GBC (*p* = 1.000, missing data recurrence location *n* = 10).

## Discussion

While it is well-known that approximately 3–15% of patients with suspected malignant hilar strictures are eventually diagnosed with benign disease after resection [1, 2], until this point no reports, to the best of our knowledge, have described the frequency and consequence of finding other types of BTC after resection for suspected pCCA. The accuracy in preoperative classification of resectable tumors is important, in light of an increased awareness of differences regarding etiology, prognosis and therapeutic response between specific types of BTC [10, 11]. The aspect of diagnostic and prognostic differentiation on preoperative staging is also of central relevance considering ongoing research in neoadjuvant therapy [12–14].

While one previous report found unresectable GBC in 14% of patients undergoing exploration for suspected hilar malignancy [15], and a second cohort with a majority of patients with unresectable tumors (65% unresectable) reported 9% of patients having GBC [16], this current study is the first to distinguish between outcomes in different types of BTC for patients with resectable suspected pCCA.

In this analysis, presenting postoperative diagnoses and short- and long-term outcomes for patients resected for suspected pCCA at a large tertiary referral center, 15% of patients were found to have GBC, defined as tumors extending from the gallbladder or cystic duct. The frequency of benign diagnosis after resection for suspected pCCA was 14%, which is in line with previous studies.

Comparing patients with a postoperative finding of GBC to patients with confirmed pCCA, patients with GBC were older, with a large proportion of tumors with high tumor extension stage, reflecting GBC tumor invasion to the hepatic hilum, with 91% of GBC patients undergoing preoperative biliary drainage due to jaundice. Importantly, the specific TNM/AJCC tumor extension categories for GBC and pCCA differ, e.g., so that extrahepatic bile duct invasion, peritoneal perforation or hepatic invasion represents a T3 extension in GBC while extension beyond the bile duct is classified as T2 in pCCA and where T3 pCCA denotes tumors with unilateral vessel involvement [9]. Furthermore, patients with GBC were found to have shorter disease-free survival.

If identified preoperatively, presence of high-risk characteristics or evidence of conditional resectability could present indications to adjust treatment sequencing for GBC patients, i.e., support a neoadjuvant strategy [17]. While overall survival was not significantly different between pCCA and GBC groups, 3-year overall survival was 33% for patients with pCCA and only 9% in patients with GBC. This is in line with recent data on the overall poor prognosis for patients with GBC [18], especially those with T3 and T4 tumors [18] and with biliary obstruction [19]. Such data has indicated the potential benefit of a neoadjuvant strategy for patients with locally advanced GBC, when identified preoperatively [18]. In a nation-wide analysis of outcomes in preoperatively identified GBC, patients with jaundice had a median overall survival of 16.7 months [19], i.e., similar to that of the patients with postoperative reclassification as GBC in the present study.

There was also evidence of less extensive surgery for patients with GBC, with significantly fewer major resections performed in this group. This could reflect the intraoperative identification of GBC rather than pCCA, as suggested in three patients operated with bisegmentectomy of segments 4 and 5. A lower rate of major resections in GBC could also represent individually tailored treatment for older patients with frailty, considering the risks associated with a major hepatectomy. To which degree the prognostic differences seen here represent differences in tumor biology or differences in surgical-oncological treatment is not clear, as the small sample size did not permit multivariable analyses. In an analysis of outcomes specifically after major right- or left-sided resections for suspected pCCA from another tertiary referral center, only one out of 178 patients (0.6%) was reclassified postoperatively as having GBC [20]. This could be both a reflection of differences in the preoperative work-up during the inclusion periods, such as the implementation of routine staging laparoscopy for Bismuth-Corlette type III and IV tumors at this center [21], and a consequence of the inclusion of only patients undergoing major hemi-hepatectomy. In a previous report including all resection types during an extended time-period (1984–2015), a similar low percentage of patients were diagnosed with GBC (0.6%) [2]. Importantly, the classification of cystic duct tumors as GBC was introduced with the 7th edition (2010) of the AJCC/TNM system [9]. Modalities suggested to have potential to contribute to the detailed preoperative evaluation of hilar lesions include cholangioscopy, intraductal ultrasound, MRI, diagnostic laparoscopy, laparoscopic ultrasound, and endoscopic ultrasound [22–25]. However, selection criteria used for an extended evaluation, as well as local expertise and availability have all been seen to influence the diagnostic value of any added investigations [22–24].

The inclusion period of the current study preceded the BILCAP trial [26] and the implementation of routine adjuvant therapy for resected BTC at this center. Even if there was no statistically significant difference in receipt of adjuvant chemotherapy, it must be noted that no patients in the GBC group received adjuvant therapy. Notably, studies on systemic chemotherapy in advanced BTC have indicated disease-specific differences in treatment response. Subgroup analysis of the ABC-02 trial, that established gemcitabine-cisplatin chemotherapy, indicated better radiological partial response for the GBC group compared to other BTC patients (partial response 37.7% vs 18.0%, no significance test reported) [27]. Pooled analyses of studies on systemic chemotherapy in BTC have shown a statistically significant difference with better radiological response in GBC, however with remaining poor survival outcomes for patients with GBC [10, 28].

One important aspect of accuracy in preoperative classification and staging is to allow the study of neoadjuvant treatment strategies. Potential benefits of a neoadjuvant strategy could be selection of surgery for patients with responsive or biologically less aggressive tumors; improved resection margin for patients with radiological response or stable disease; and a better chance of completion of both local and systemic therapy when systemic treatment is given first, especially if local therapy has a high risk of morbidity. The poor long-term prognosis and considerable rate of major complications and 90-day postoperative mortality after surgery for pCCA underscores the importance of understanding the prognostic impact of preoperatively available factors [29–31].

A recent systematic review of retrospective cohort studies of neoadjuvant therapy in GBC showed a pooled resection rate of 52%, R0 rates ranging from 25 to 100% and median overall survival ranging from 18.5 to 50.1 months for patients with ≥ stage IIIa GBC resected after neoadjuvant therapy [32]. The R1 rate for GBC patients resected with a preoperative diagnosis of pCCA in the current study was 73%, and 91% of patients had T3 or T4 tumors. More than 60% of GBC patients suffered a severe complication and postoperative mortality in this subgroup was 18%. Whether a neoadjuvant strategy could improve outcomes for older BTC patients with locally advanced tumors needs to be studied in prospective trials. In pancreatic cancer, conditional criteria including performance status and biological factors such as an increased GPS have been proposed as potential indications for a neoadjuvant strategy [33, 34]. With regard to preoperative immune-related risk factors, no difference was seen in the rates of increased GPS score between the GBC and pCCA groups in this study. The overall R1 rate of 85% for patients with GBC or verified pCCA in this study was reported according to the institutional definition of R0 as a tumor-free microscopic margin of 1 mm or more [8].

This study needs to be seen in the light of several limitations. Firstly, with a single-center setting, the sample size was limited. In addition, while relatively recent, the study period preceded the BILCAP trial [26] and few patients received adjuvant chemotherapy. Furthermore, with a retrospective design, selection and information bias may occur. However, the clinical management remained uniform over the study period, and multidisciplinary tumor board diagnosis was reported. Lastly, the study lacks any data to compare outcomes of patients reclassified as having GBC with those of patients with a proper preoperative diagnosis of GBC receiving curative intent resection.

## Conclusions

In this cohort study from a large tertiary referral center, 15% of patients resected for suspected pCCA were postoperatively diagnosed with GBC. Compared to patients with pCCA, GBC patients were older, had a high risk of advanced tumors and shorter disease-free survival. The considerable rate of re-classification stresses the need for improved preoperative staging, as these prognostic differences could have implications for the therapy strategy.

## Data Availability

The data supporting the findings of this study are available from the corresponding author on reasonable request.

## Abbreviations

ASA: American Society of Anesthesiologists physical status classification
BMI: Body mass index
BTC: Biliary tract cancer
CCA: Cholangiocarcinoma
CT: Computerized tomography
DFS: Disease-free survival
ERC: Endoscopic retrograde cholangiography
GBC: Gallbladder cancer
GPS: Glasgow prognostic score
IQR: Interquartile range
LV1: Lymphovascular invasion
MRI: Magnetic resonance imaging
N1: Lymph node metastasis
pCCA: Perihilar cholangiocarcinoma
PSC: Primary sclerosing cholangitis
PTC: Percutaneous transhepatic cholangiography
PVE: Portal vein embolization
R1: Microscopically positive resection margin
T ≥ 3: Tumor extension 3–4
